# Breaking the blue spectral barrier of non-conjugated hydrocarbons

**DOI:** 10.1093/nsr/nwag469

**Published:** 2026-08-03

**Authors:** Jungryun Kim, Eunji Kim, Jaewon Kim, Kippeum Lee, Yuling Xu, Min-Goo Lee, Jong Seung Kim

**Affiliations:** Department of Chemistry, Korea University, Korea; Department of Chemistry, Korea University, Korea; Department of Chemistry, Korea University, Korea; Department of Chemistry, Korea University, Korea; Department of Chemistry, Korea University, Korea; Department of Chemistry, Korea University, Korea; Department of Chemistry, Korea University, Korea; National Research Laboratory for Convergence Degradation Biology, Korea University, Korea

The luminescence of organic molecules has long been governed by the paradigm of through-bond conjugation (TBC), in which extended π-electron delocalization is considered essential for long-wavelength emission. This principle has consigned non-conjugated systems, particularly pure hydrocarbons, to the role of non-emissive background materials [1]. Ben Zhong Tang, Haoke Zhang, and co-workers developed the through-space interaction (TSI) strategy as a compelling alternative for constructing transformative luminescent materials, demonstrating that visible fluorescence can arise from spatially proximal but covalently disconnected chromophores [2,3]. However, a significant bottleneck persists: the fluorescence of non-conjugated hydrocarbons has largely been confined to the blue region, raising a fundamental question about the intrinsic limits of this new photophysical mechanism.

To address this challenge, Zheng Zhao (CUHK-Shenzhen) and Xiaoming Wang (SIOC), together with their co-workers, recently reported a remarkable breakthrough in extending emission wavelength, shattering the ‘blue limit’ [4]. By constructing a library of more than 50 non-conjugated arylated alkenes via a binuclear nickel-catalyzed hydroarylation of allenes (Fig. 1A), the authors demonstrate that pure hydrocarbon systems can be engineered to emit across a broad spectrum, from blue through yellow and orange, and even produce warm white light from a single compound.

**Figure 1.**
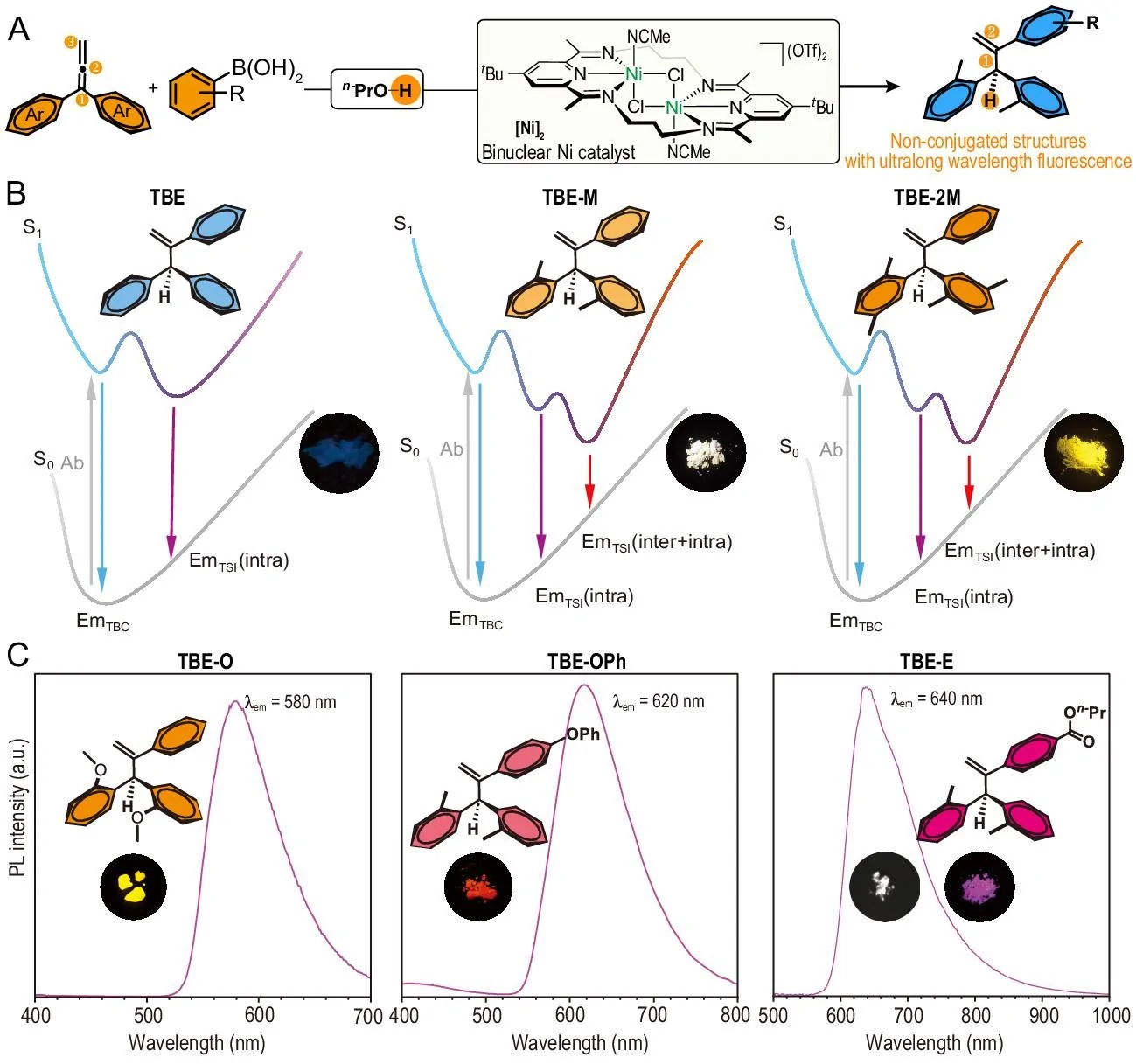
(A) Synthesis of non-conjugated molecules via dinickel-catalyzed hydroarylation of allenes. (B) Proposed luminescence mechanism and potential energy surfaces of TBE derivatives (TBE, TBE-M and TBE-2M) in the aggregate. (C) Photoluminescence (PL) intensity spectra of TBE derivatives (TBE-O, TBE-OPh and TBE-E) in the solid state. Adapted from Ref. [4] under the Creative Commons Attribution 4.0 International License (CC BY 4.0). Copyright © 2026, The Author(s). Changes were made.

The key to this achievement lies in the synergistic interplay among multiple TSIs. The team meticulously elucidated that intramolecular TSIs, tuned by molecular twisting, combine with intermolecular TSIs mediated by the double bonds of styrene units. This synergy creates a new, effective chromophore in the aggregate state.

Crucially, they identified that intermolecular C=C···C=C interactions between adjacent styrene units serve as the primary bridge for electron delocalization, with shorter distances directly correlating to longer emission wavelengths (Fig. 1B). By strategically modifying the styrene bridge, the authors further extended the emission into the deep-red region, with an emission tail detectable beyond 900 nm (Fig. 1C).

Beyond the experimental observations, the authors provide compelling theoretical evidence from ONIOM calculations and IGMH analysis, revealing that methyl substitution induces molecular frustration that counterintuitively enhances C=C···C=C interactions despite increased steric hindrance. This structural perturbation facilitates through-space electron delocalization in dimers in the aggregate state, giving rise to hybrid local-excited and charge-transfer characteristics that underpin the redshifted emission. The tunability of this design principle is further validated by the successful extension of the emission palette through simple functional-group modifications on the styrene unit, demonstrating the robustness and tunability of the TSI-based strategy.

This work advances our understanding of luminescence in non-conjugated systems by showing that the absence of extended TBC does not necessarily preclude long-wavelength emission. It establishes a novel design strategy for luminescent materials based on TSIs, offering a sustainable, synthetically accessible alternative to traditional conjugated frameworks and promising implications for optoelectronics and bioimaging [5]. The combination of facile synthesis, structural simplicity, and broadly tunable emission positions these non-typical luminogens as attractive candidates for next-generation sensing and imaging applications.
